# Selection and validation of reference genes for quantitative gene expression studies by real-time PCR in eggplant (*Solanum melongena* L)

**DOI:** 10.1186/1756-0500-6-312

**Published:** 2013-08-06

**Authors:** Nagavara Prasad Gantasala, Pradeep Kumar Papolu, Prasoon Kumar Thakur, Divya Kamaraju, Rohini Sreevathsa, Uma Rao

**Affiliations:** 1 Division of Nematology, Indian Agricultural Research Institute, New Delhi, 110012, India; 2 Department of Genetic Engineering, SRM University, Chennai, India; 3 National Research Centre for Plant Biotechnology, IARI, Pusa Campus, New Delhi, India

**Keywords:** Reference genes, Housekeeping genes, Eggplant (or) brinjal, *Solanum melongena L*, Quantitative real-time PCR (qRT-PCR), Normalization, Gene expression

## Abstract

**Background:**

Analysis of gene expression patterns leads to functional understanding of biological processes. Quantitative real-time PCR has become the most commonly used technique for in-depth studies of gene expression. To quantify variation in specific gene expression, accurate and reliable normalization across different samples and tissues is necessary. This can be achieved by selecting one or more suitable reference genes to compare the target mRNA transcript levels. In the present work, we illustrate the first evaluation of potential internal control or reference genes across different developmental stages of eggplant for reliable quantification of transcripts by real-time PCR.

**Results:**

We have evaluated the stability in expression of six candidate reference genes (*18s rRNA, APRT, GAPDH, Cyclophilin, Actin*, and *RuBP*) in a set of tissues representing six developmental stages of eggplant. The candidate genes were cloned from cDNA and analysed by real-time PCR. The expression data analyzed by three statistical methods (geNorm, NormFinder and BestKeeper) identified *18s rRNA, Cyclophilin* and *APRT* as the most stable and suitable reference genes in eggplant. This was further confirmed in four different varieties, two representative lines of transgenic eggplant as well as in nematode infected eggplant.

**Conclusion:**

*18s rRNA, Cyclophilin* and *APRT* have been found to be appropriate for the normalization of real-time PCR data for gene expression studies in eggplant.

## Background

Gene expression analysis is an important tool to understand the functional aspect of genes. Quantification of the steady-state mRNA by real-time PCR is identified as the most reliable and commonly used approach for basic research, molecular medicine and biotechnology [1,2]. Gene expression levels are routinely evaluated using approaches such as, northern hybridization and reverse transcription-polymerase chain reaction (RT-PCR). The possibility of high throughput analysis combined with its high sensitivity, reliable specificity and simplicity render the real-time PCR approach as the most appropriate strategy [3-5]. However, the real-time PCR based approach can be dependable only when the results are normalized. The use of unreliable reference genes or internal control genes for normalization of the data is the major lacuna of this approach [6,7]. Normalization is essential for correcting the errors that could arise due to inaccurate quantification of RNA and problems in the quality of RNA leading to variable reverse transcription and PCR reactions. Even though various strategies are employed to normalize qRT-PCR (quantitative-real time PCR), it still remains as one of the primary challenges in the utility of this technique [7]. A gene whose expression remains stable across tissues and developmental stages would represent the best system for normalization of the qRT-PCR data. The stability of such reference genes across genotypes of a particular crop species would be an added advantage.

Some of the house keeping genes involved in basic cellular activities such as *18s rRNA*, *25s rRNA*, glyceraldehyde-3-phosphate dehydrogenase (*GAPDH*) and *ubiquitin* (*UBQ*) are some of the commonly used internal control genes as they are likely to be expressed at constant levels regardless of experimental conditions [8-12]. However, recent studies suggested that expression of these genes vary significantly under different environmental conditions [6,13,14]. Therefore, it is necessary to identify the internal control gene (s) for the efficient quantification of a target mRNA by qRT-PCR in a given set of biological samples. Several statistical algorithms such as geNorm, BestKeeper and NormFinder have been developed to determine the stability of reference genes in a given set of biological samples [15,16]. Several studies have used these programs for the evaluation of various housekeeping genes to establish their utility as reference genes for normalization of real-time PCR data in various plants [8,14,17-20].

Eggplant popularly known as Brinjal in India, is an agronomically important non-tuberous Solanaceous crop primarily grown for its large oval fruit. In addition to being a popular vegetable, it has therapeutic value and used for the treatment of several diseases such as diabetes, arthritis, asthma and bronchitis [21]. It can be a good alternate model plant for studying various agronomic traits through transgenic technology because of its high response to *in vitro* regeneration leading to high genetic transformation efficiency [22]. Genomics efforts have lead to the accumulation of approximately 98,089 ESTs in eggplant (NCBI dbEST) which can be an excellent source for prospecting novel genes and deciphering their biological functions. However, gene expression analysis by qRT-PCR is limited in eggplant primarily due to the lack of information about genes which can serve as internal controls. In this context, the present study was undertaken to select and validate the most suitable internal control gene(s) in eggplant for effective normalization of the qRT-PCR data.

## Results

### Cloning of the reference genes from cDNA of eggplant

**Figure 1:**
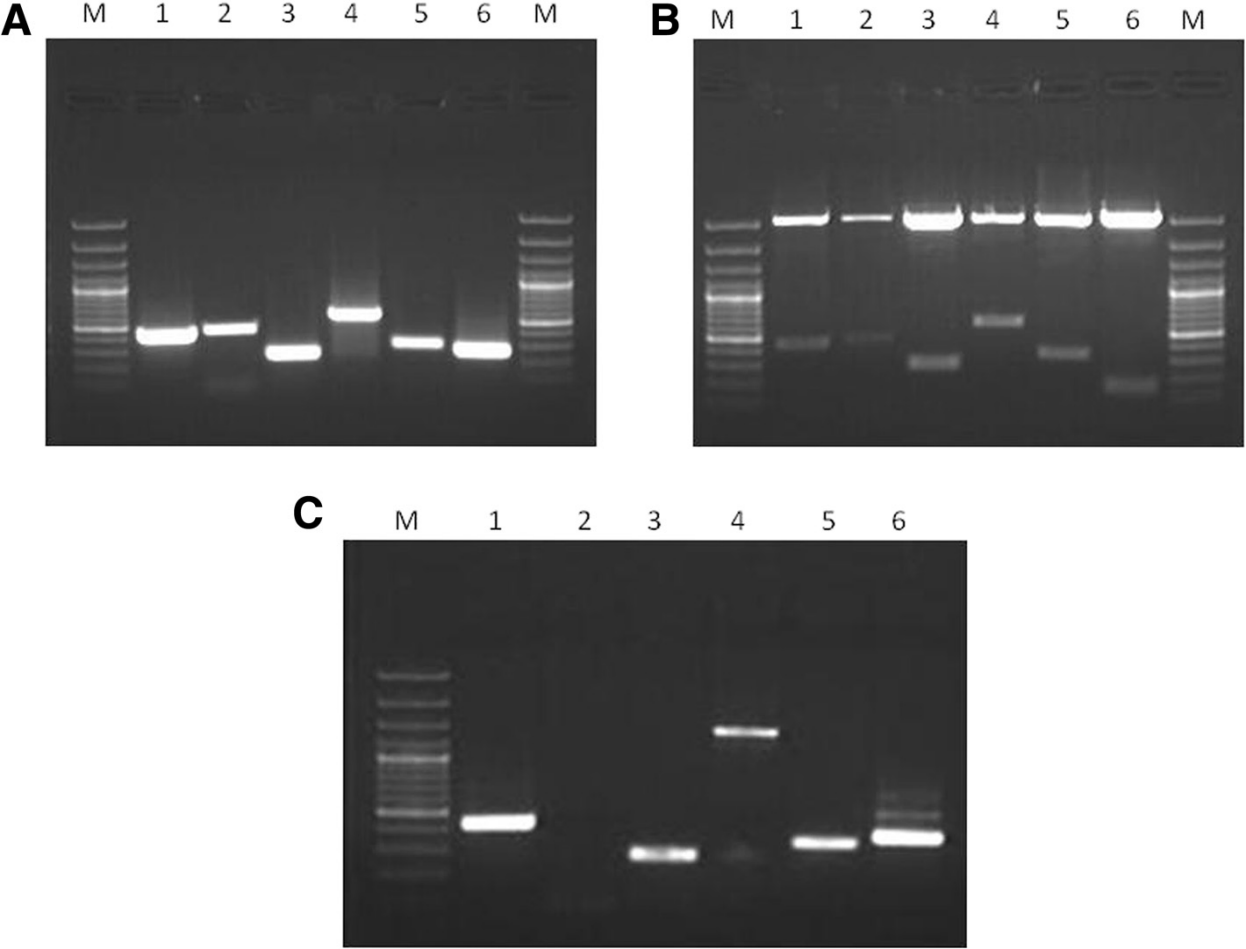
**Amplification of the housekeeping genes and confirmation of cloning by *EcoRI *digestion of pGEM-T vector. (A)** Amplification of the six target genes from cDNA of eggplant Lanes - M: 100 bp DNA Ladder/Marker, 1. *18S rRNA* (416bp), 2: *APRT* (454 bp), 3: *GAPDH* (586bp), 4: *Cyclophilin* (265 bp), 5: *Actin* (333 bp), 6: *RuBP* (269bp). **(B)** Confirmation of the inserts in the recombinant pGEM-T vector by *EcoRI* digestion. Lanes - M: 100 bp DNA Ladder/Marker, 1. *18S rRNA* (416bp), 2: *APRT* (454 bp), 3: *Cyclophilin* (265 bp), 4: *GAPDH* (586bp), 5: *Actin* (333 bp), 6: *RuBP* (269bp). **(C)** Amplification of the six target genes from genomic DNA of eggplant. Lanes- M: 100 bp DNA Ladder/Marker, 1. *18S rRNA* (416bp), 2: *APRT* (No amplification), 3: *Cyclophilin* (265 bp), 4: *GAPDH* (1.2 kb), 5: *Actin* (333 bp), 6: *RuBP* (269bp).

**Figure 2:**
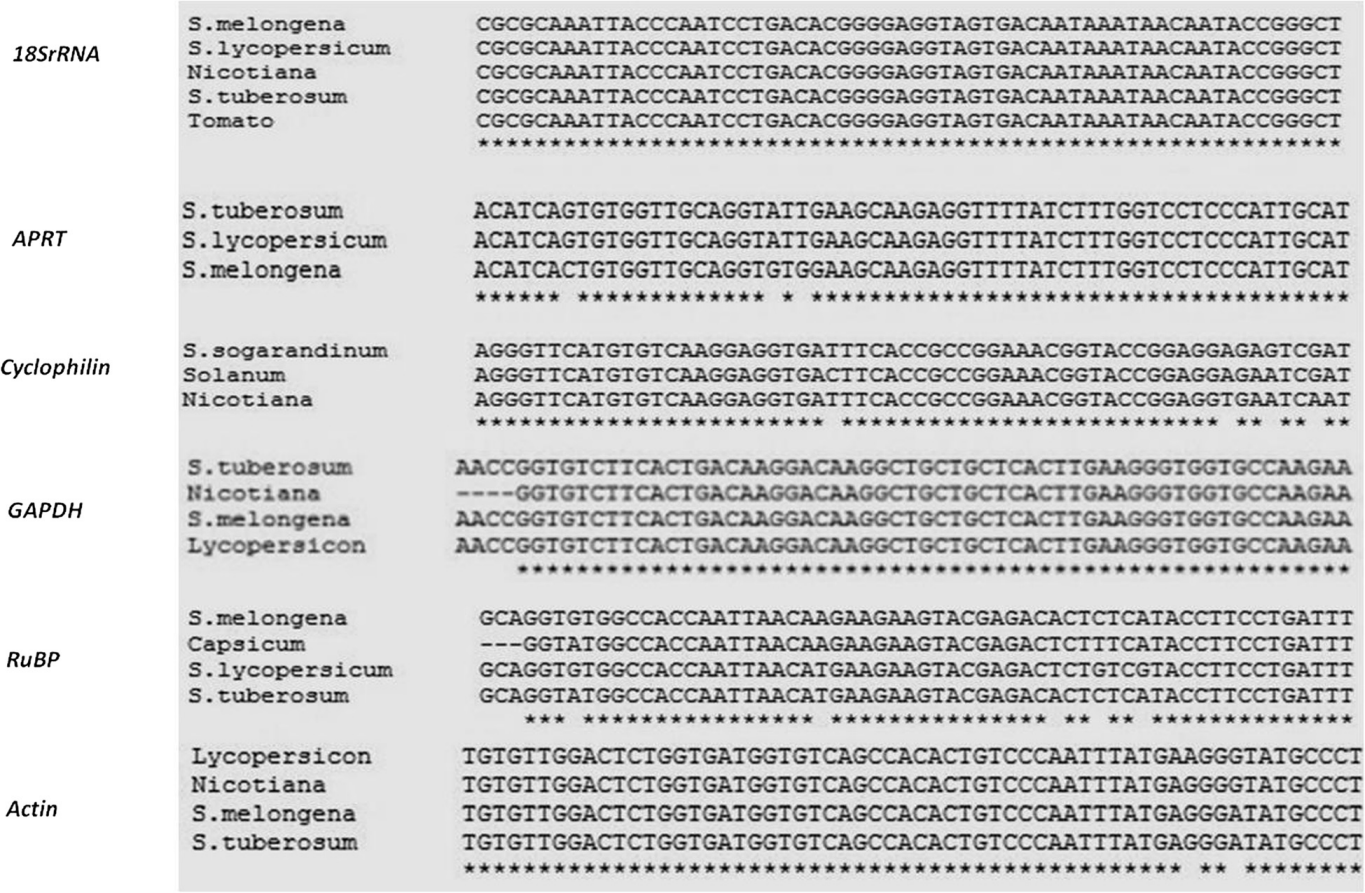
Multiple Sequence Alignment of the six candidate genes by CLUSTALW.

PCR amplification of six target reference genes viz., *18s rRNA*, *APRT, GAPDH, Cyclophilin, Actin, RuBP* from the cDNA resulted in 416 bp, 454 bp, 586 bp, 265 bp, 333 bp, and 269 bp amplicons respectively, which were cloned and sequenced (Figure 1A &1B). Moreover comparative analysis of the sequenced products revealed high (95-100%) similarity with the members of *Solanaceae* family (Figure 2 &Table 1). The sequences were later deposited in the GenBank database (GenBank: JX448341, JX448342, JX448343, JX448344, JX448345, JX524155). In order to check the presence of introns between the primer binding sites, all the six reference genes were PCR amplified from the genomic DNA extracted from the eggplant leaves (Figure 1C). Except for *APRT*, amplification was successful for all the other five genes. There was no amplification in *APRT* probably due to the presence of a big intronic region; in case of *GAPDH*, the amplicon was bigger than the cDNA fragment indicating the presence of an intron. With respect to the other four genes, the size of the genomic DNA fragments was same as that of the cDNA fragments.

**Table 1:** BLAST analysis of the reference gene sequences

**Gene name**		**Accession**	**Query coverage**	**E value**	**Max ident**
*18S rRNA*	*Solanum melongena*	JX448341	100%	0.0	100%
	*Solanum tuberosum*	X67238	100%	0.0	100%
	*Solanum lycopersicum*	AC246968	100%	0.0	98%
	*Solanum lycopersicum*	X51576	100%	0.0	98%
	*Nicotiana tabacum*	HQ384692	100%	0.0	97%
*APRT*	*Solanum melongena*	JX448345	100%	0.0	100%
	*Solanum tuberosum*	DQ284483	100%	0.0	94%
	*Solanum lycopersicum*	AK321573	95%	1e-176	92%
*GAPDH*	*Solanum melongena*	JX448342	100%	0.0	100%
	*Solanum tuberosum*	AF527779	100%	0.0	97%
	*Solanum lycopersicum*	U97257	100%	0.0	96%
	*Nicotiana langsdorffii*	EF636821	99%	0.0	95%
*Cyclophilin*	*Solanum melongena*	JX448344	100%	2e-137	100%
	*Solanum tuberosum*	DQ235183	100%	2e-107	93%
	*Solanum sogarandinum*	EF043281	100%	4e-104	92%
	*Nicotiana tabacum*	AY368274	100%	4e-104	93%
*Actin*	*Solanum melongena*	JX524155	100%	4e-175	100%
	*Solanum tuberosum*	X55749	100%	4e-175	100%
	*Solanum lycopersicum*	BT012695	99%	9e-122	91%
	*Nicotiana tabacum*	AF154640	99%	2e-114	89%
*RuBP*	*Solanum melongena*	JX448343	100%	1e-139	100%
	*Solanum lycopersicum*	AK319576	95%	7e-102	93%
	*Capsicum annuum*	AF065615	95%	9e-96	91%
	*Solanum tuberosum*	JX576219	95%	7e-92	90%

### Assessment of expression stability of reference genes

**Figure 3:**
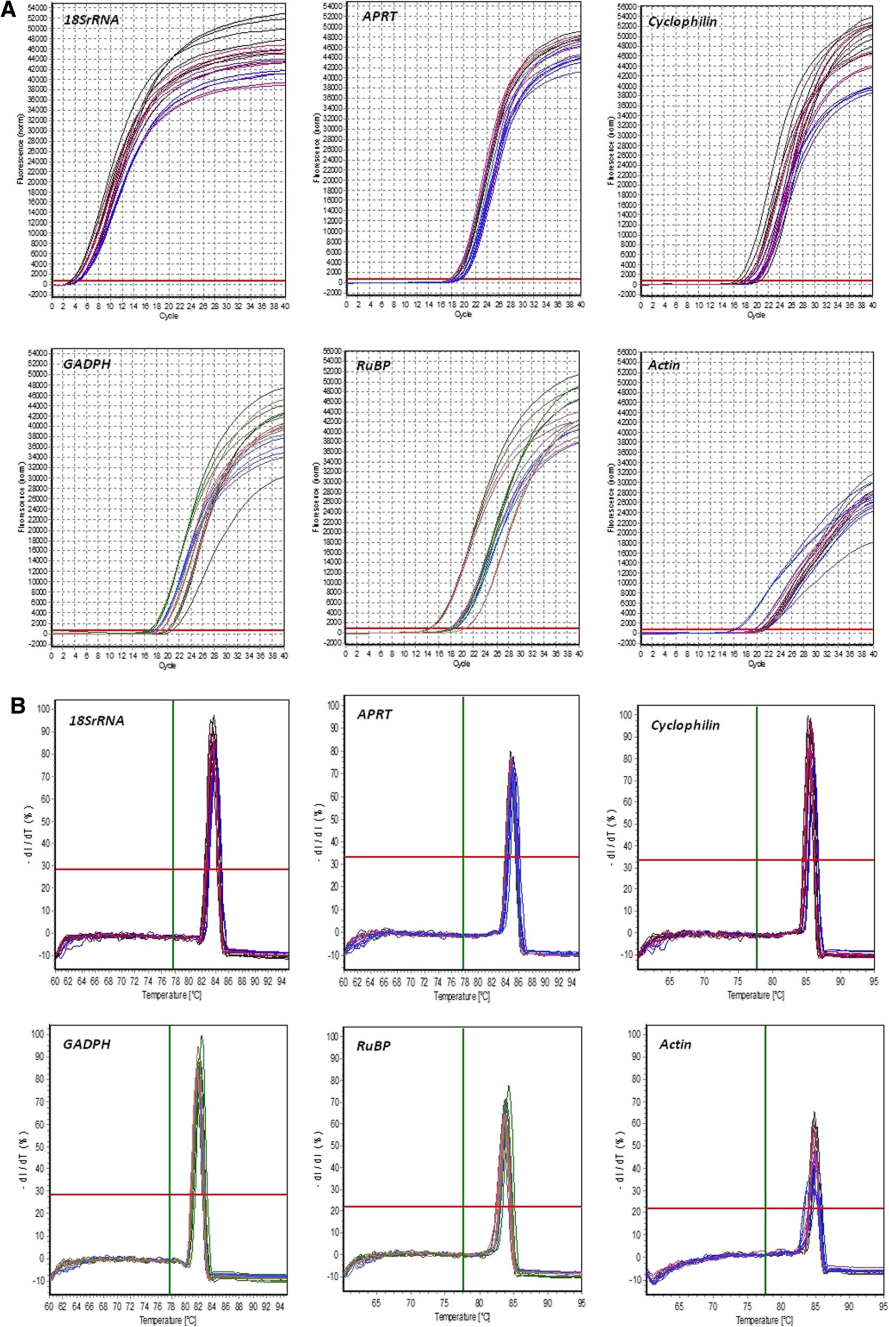
Specificity of real-time PCR amplification (A) Quantification curve for the six candidate reference genes from cDNA of six different tissues (B) Melting curve for the six candidate reference genes from cDNA of six different.

Genes encoding for *18s rRNA*, adenine phosphoribosyl transferase (*APRT*), glyceraldehyde 3-phosphate dehydrogenase (*GAPDH*), *Cyclophilin*, *Actin*, Ribulose-1,5-bisphosphate carboxylase (*RuBP*) were selected based on previous studies that relied on them as candidate reference genes [23-25]. In order to calculate the stability of expression of the selected six candidate reference genes, mRNA expression levels were measured in six different tissues of the eggplant (young leaf, mature leaf, shoot, root, flower bud and open flower). Ct mean values of three biological replicates were obtained from Realplex^2^ software. These Ct mean values were further used for the calculation of expression stability (Figure 3A &3B, Table 2). Real-time PCR analysis revealed a large significant similarity in the observed expression pattern of all the genes across various tissues. Three commonly used statistical algorithms *viz*., BestKeeper, Normfinder and geNorm were employed for normalization of expression pattern and to validate Ct values for choosing the best reference genes.

**Table 2:** Expression pattern of the reference genes in different tissues of eggplant

**Cq ± SD**						
**Plant tissue**	***18S rRNA***	***APRT***	***GAPDH***	***Cyclophilin***	***Actin***	***RuBP***
Young leaf	4.26 ± 0.26	19.16 ± 0.05	20.19 ± 0.61	19.95 ± 0.66	20.85 ± 0.48	15.03 ± 0.27
Mature leaf	4.16 ± 0.05	19.17 ± 0.23	19.73 ± 0.07	20.95 ± 0.19	21.16 ± 0.43	14.77 ± 0.09
Shoot	4.41 ± 0.06	18.11 ± 0.05	17.91 ± 0.05	21.95 ± 0.27	20.99 ± 0.49	18.47 ± 0.31
Root	4.35 ± 0.20	17.91 ± 0.04	17.65 ± 0.04	22.95 ± 0.48	21.18 ± 0.20	18.93 ± 0.12
Flower bud	3.35 ± 0.13	17.52 ± 0.03	16.80 ± 0.02	23.95 ± 0.69	19.57 ± 0.09	18.17 ± 0.29
Open flower	3.80 ± 0.03	18.69 ± 0.15	18.69 ± 0.11	24.95 ± 0.16	16.37 ± 0.17	21.11 ± 0.45

### Best keeper analysis

**Figure 4:**
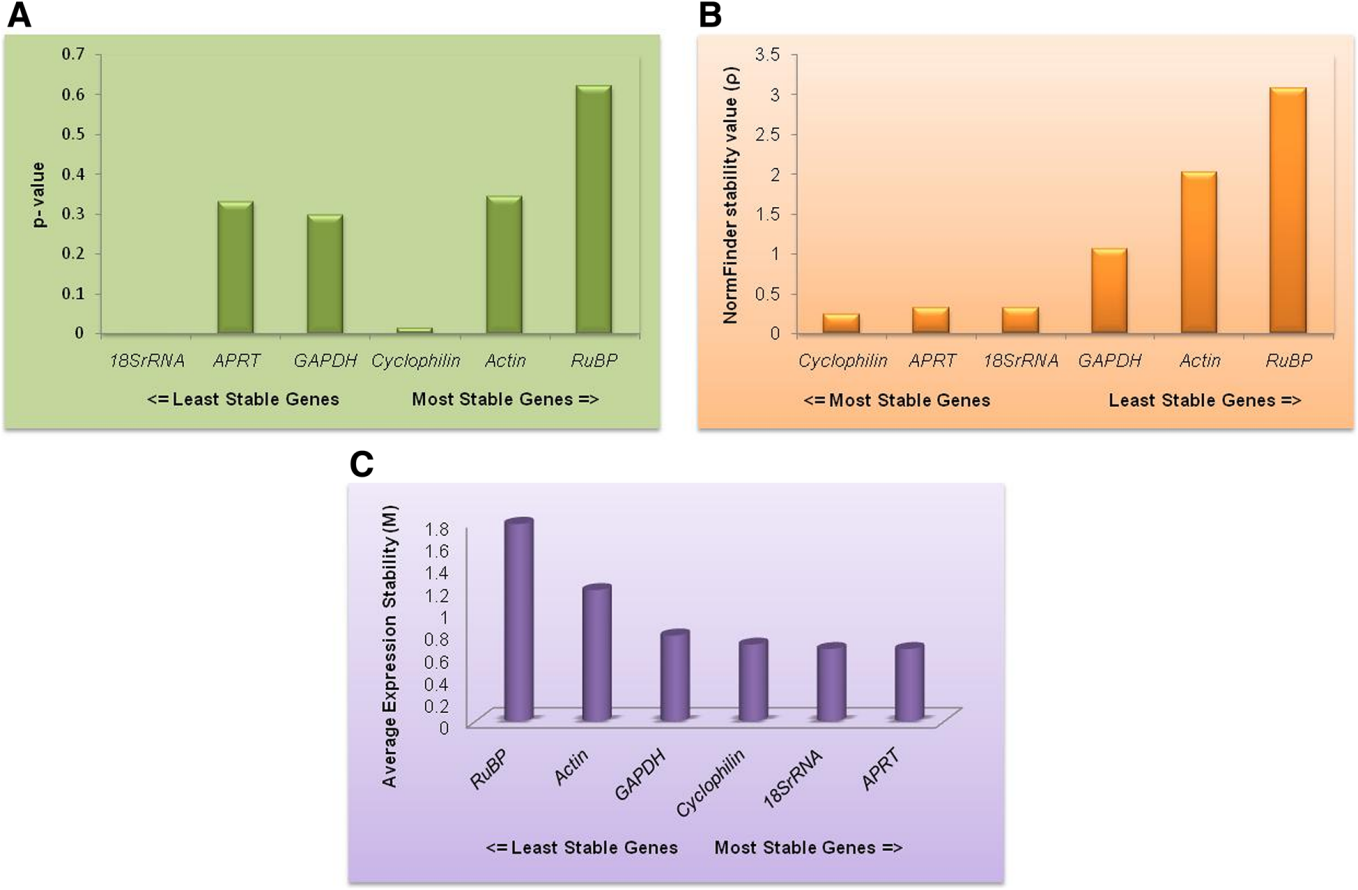
Expression stability and ranking of housekeeping genes by three statistical softwares, (A) Best keeper (B) NormFinder (C) geNorm.

This analysis was done using the raw Ct values. Initially, variations (SD (± Ct) and CV (%Ct)) were calculated for each of the candidate reference genes in the samples for identifying the overall stability in gene expression. Three candidate reference genes (*GAPDH*, *Actin* and *RuBP*) showed an SD value higher than 1, which disqualified their utility as reference genes. Remaining three genes were selected for further analysis as they showed SD value less than 1. Further processing of the data using pair wise correlation and regression analysis showed the inter gene relations and eliminated *APRT*, as the gene with the least correlation (r = 0.481) (Table 3). The analysis of the remaining two genes (*18s rRNA* and *Cyclophilin*) showed a strong and significant correlation with an ‘r’ value of 0.990 for *18S rRNA* and 0.895 for *Cyclophilin* indicating their stable expression levels. Accordingly, the BestKeeper index (p) was found to be 0.001 and 0.016 respectively for *18s rRNA* and *Cyclophilin* (Figure 4A). In view of high correlation value and low BestKeeper index, *18s rRNA* and *Cyclophilin* were selected as the two likely reference genes.

**Table 3:** Statistical analysis of the expression data of six housekeeping genes

**Gene name**	**BestKeeper coefficient**	**NormFinder stability**	**geNorm expression**
	**of correlation (r)**	**value (ρ)**	**stability (M)**
*18S rRNA*	0.990	0.3245	0.648981
*Cyclophilin*	0.895	0.2342	0.688693
*APRT*	0.481	0.314	0.648981
*GAPDH*		1.0646	0.770861
*Actin*		2.0171	1.181311
*RuBP*		3.0834	1.776904

### NormFinder analysis

NormFinder analysis results revealed that the gene expression of three candidate reference genes, *Cyclophilin*, *APRT* and *18SrRNA* had lower stability values across the six tissue samples (Table 3). Further, manual inspection of the remaining reference genes showed that *GAPDH* ranked at the fourth position, had higher intra-group variation than *18s rRNA*. Nevertheless, *Actin* and *RuBP* had highest intra-group variation and highest stability values. Thus, based on NormFinder analysis, *Cyclophilin*, *APRT* and *18s rRNA* were identified as the best candidate reference genes (Figure 4B).

### geNorm analysis

Analysis of raw non-normalized data of six different tissue samples (n = 6) allowed sorting of genes ranked on the basis of their expression stability (M) from most stable to least stable in the order of *APRT*, *18s rRNA*, *Cyclophilin*, *GAPDH*, *Actin* and *RuBP*. Their calculated ‘M’ values were 0.648981, 0.648981, 0.68869, 0.770861, 1.18131 and 1.77690 respectively (Table 3, Figure 4C). Eventually, successive elimination of the least stable genes based on the highest ‘M’ values led to the identification of *APRT* and *18s rRNA* as the two potential reference genes.

Therefore, based on the elaborate statistical analysis *18s rRNA*, *APRT* and *Cyclophilin* were identified as appropriate reference genes.

### Validation of reference genes

The selected genes were analyzed under various situations to assess their utility to serve as reference genes:

#### Varietal influence on the expression of identified genes

**Figure 5:**
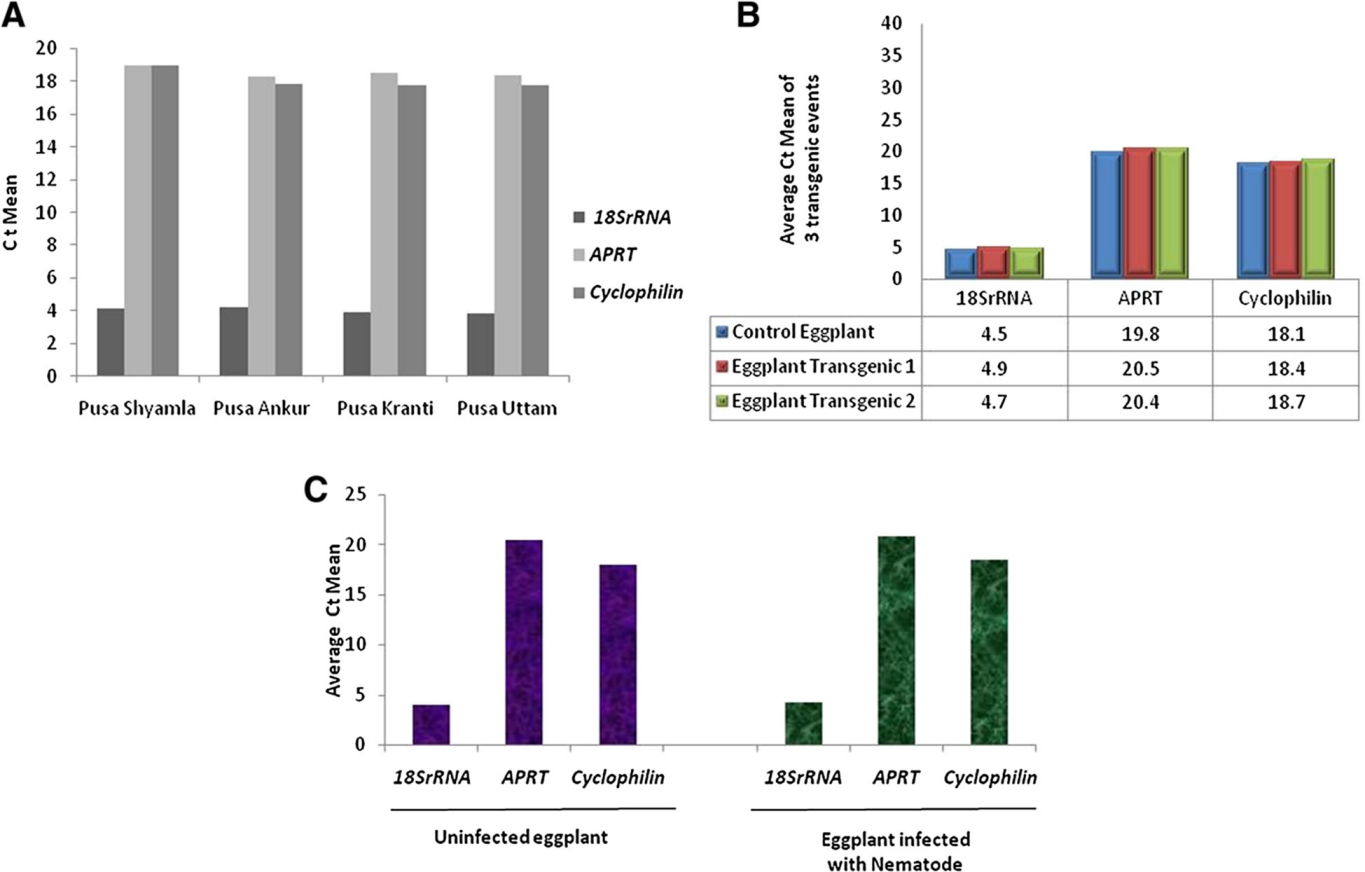
Validation of reference genes (A) Validation in other eggplant varieties (B) Validation in transgenic eggplant (C) Validation in the presence of nematode stress.

The utility of the identified genes was further analyzed in four different eggplant varieties using real-time PCR (Table 4). An expression pattern of high similarity was observed in all the four genotypes with every selected gene. This demonstrated the utility of the selected genes as reference genes for real-time PCR analysis in eggplant (Figure 5A).

**Table 4:** Confirmation of the stability of the identified reference genes in four different varieties of eggplant

**Cq ± SD**			
**Name of variety**	18S rRNA*	APRT*	Cyclophilin*
Pusa Shyamla	4.15 ± 0.14	18.99 ± 0.24	19.04 ± 0.40
Pusa Ankur	4.24 ± 0.02	18.36 ± 0.01	17.86 ± 0.12
Pusa Kranti	3.95 ± 0.01	18.55 ± 0.03	17.78 ± 0.00
Pusa Uttam	3.88 ± 0.01	18.38 ± 0.00	17.80 ± 0.04

*Expression analysis of the three housekeeping genes and average values of quantification cycle (Cq) ± standard deviation (SD) of biological replicates of four different eggplant varieties.

#### Utility of the identified genes in transgene expression analysis

Further to the validation of the selected genes in different eggplant varieties, their utility was also established in the analysis of transgenics (Table 5, Figure 5B). The reference genes play a vital role in the expression analysis of the transgenics using real-time PCR. Real-time analysis of two representative transgenic lines revealed similar expression pattern of all the three housekeeping genes.

**Table 5:** Confirmation of expression stability of the reference genes in representative transgenic eggplants

**Cq ± SD**			
**Plants**	***18S rRNA***	***APRT***	***Cyclophilin***
**Control**	4.50 ± 0.25	19.80 ± 0.20	18.10 ± 0.30
**Transgenic1**	4.91 ± 0.16	20.50 ± 0.23	18.47 ± 0.14
**Transgenic 2**	4.72 ± 0.15	20.48 ± 0.14	18.78 ± 0.20

#### Expression analysis of the identified genes in eggplant under nematode stress

The expression of the selected reference genes was also validated in the plants that were challenged with root knot nematodes (*Meloidogyne incognita*) (Table 6, Figure 5C). It was observed that the expression of the three reference genes in the leaf tissue of the nematode challenged plants was similar to that of the uninfected plants.

**Table 6:** Confirmation of expression stability of the reference genes in nematode infected eggplants

**Cq ± SD**			
**Plants**	***18S rRNA***	***APRT***	***Cyclophilin***
**Control**	4.05 ± 0.10	20.42 ± 0.30	18.05 ± 0.17
**Nematode infected**	4.20 ± 0.26	20.85 ± 0.28	18.50 ± 0.19

These above analyses ultimately confirmed the identification and utility of the three housekeeping genes as the appropriate reference genes for qRT-PCR analysis of gene expression in eggplant.

## Discussion

Gene expression profile studies provide an important insight into the biological processes of plant molecular biology research, hence are recognized as crucial steps in identifying gene function. Among the various techniques available to quantify gene expression, qRT-PCR is the most commonly used method [26-28]. It is the only sensitive technique available to measure the quantity of low abundance proteins [7,13]. However, to get dependable results from real-time PCR analysis, accurate normalization of gene expression against a reference gene is required. The reference gene should have stable expression independent of the experimental conditions, developmental stages, tissues etc. However, it is very difficult to get a single gene that can qualify to be an ideal reference gene for gene expression analysis. Inappropriate use of reference genes would lead to a biased gene expression analysis. Hence, an appropriate internal control gene is required for reliable quantification of gene transcripts [26]. Further, there is a need to identify and also confirm them for a given plant species as the reference genes authenticated in one organism or plant or a biological system may not be suitable for gene expression analysis in another plant. For example, *UBQ10* gene shows highly stable expression in *Arabidopsis*[8], but is not suitable for normalization in different tissues at different developmental stages in rice and soybean [25,29]. So far, such validated reference or internal control genes have not been reported in eggplant which could pose a limitation for undertaking gene expression studies in this important species. The primary focus of the present study was to identify and validate internal control genes from eggplant that can be used for normalizing the qRT-PCR data. In this direction, six housekeeping genes viz., *18s rRNA*, *APRT*, *GAPDH*, *Cyclophilin*, *Actin* and *RuBP* were selected and their expression examined across six developmental stages of eggplant variety, Pusa Purple Long. In order to minimize bias introduced by the validation approach, the results were analyzed by three different complementary statistical strategies to select best internal controls for normalization of gene expression studies. Best keeper selects the least variable gene using the geometric mean of the raw data [15]. NormFinder not only measures the variation but also ranks the potential reference genes by how they differ between the studies [2] and geNorm allows the most appropriate mean of the expression of the candidate cDNA [25]. Using the three standard statistical analyses, *18s rRNA, APRT* and *Cyclophilin* were identified as appropriate genes for normalization. Nevertheless, earlier studies revealed that the expression of both *18s rRNA* and *Cyclophilin* were affected by both biotic and abiotic stress in potato [23]. On the other hand, *18s rRNA* exhibited most stable expression in plants grown under various environmental conditions in rice [25]. Interestingly, *GAPDH* was found to be a suitable reference gene for measuring the gene expression in different tissues/organs of sugarcane [30]. A report in *Brassica juncea*[31] revealed the combination of *GAPDH* with three other genes as suitable reference genes across different developmental stages.

Major crux of the investigation was to demonstrate the utility of the identified genes for expression analysis under different experimental conditions. The study successfully provided evidence in establishing stable expression of the identified genes across different eggplant varieties. The expression of the genes was stable in different transgenic plants and also under biotic stress deliberately induced by challenging with nematodes. This provided an unequivocal evidence for the utility of these identified genes for expression analysis by qRT-PCR in eggplant.

## Conclusion

We have cloned, sequenced and identified three genes viz., *18s rRNA*, *APRT* and *Cyclophilin* as reference genes in eggplant, suitable for normalizing real-time PCR data. This is the first study to identify the appropriate reference genes in eggplant. With the growing genomic resources in eggplant, these genes would enable accurate and reliable gene expression data analysis over a wide range of samples/experimental conditions for functional genomics as well as in translational research.

## Methods

### Plant material and growth conditions

Eggplant seeds (CV. Pusa Purple Long) were procured from National Seeds Corporation Limited, Indian Agricultural Research Institute (IARI), New Delhi. Seeds were germinated and plants were grown under controlled conditions. Young leaves, stems and roots were collected for analysis at 20 days, while flower buds, open flowers and mature leaves were collected after 45–50 days. Three independent biological replicates were collected for each of the samples and immediately frozen in liquid nitrogen.

### Primers

Nucleotide sequences of six housekeeping genes; *18s rRNA*, adenine phosphoribosyl transferase (*APRT*), glyceraldehyde 3-phosphate dehydrogenase (*GAPDH*), *Cyclophilin, Actin* and Ribulose-1,5-bisphosphate carboxylase (*RuBP*) were obtained from the GenBank database. Using primer quest tool in Integrated DNA Technology website, primers were designed for these sequences to be used in real-time PCR (200 bp maximum length, optimal Tm at 60°C, GC content of 45-50%). Details of the genes and their primers for both cDNA amplification and quantification by real-time PCR are given in Tables 7 and 8 respectively.

**Table 7:** Details of primers used for the cDNA amplification of six candidate reference genes

**Name**	**Accession number**	**Forward primer 5′-3′**	**Reverse primer 5′-3′**	**Length (bp)**	**Tm(°C)**
*18S rRNA*	X67238	CGCGCAAATTACCCAATCCTGACA	TCCCGAAGGCCAACGTAAATAGGA	416	60
*APRT*	CK270447	TGGCGCCTCATGATCCGATTCTTA	ACTCCAACACGCTCAAGAAGCCTA	454	60
*GAPDH*	U17005	AACCGGTGTCTTCACTGACAAGGA	GCTTGACCTGCTGTCACCAACAAA	586	60
*Cyclophilin*	AF126551	AGGGTTCATGTGTCAAGGAGGTGA	TCCAACAGCCTCGGCCTTCTTAAT	265	60
*Actin*	X55749	TGTGTTGGACTCTGGTGATGGTGT	AATAGGACCTCAGGGCAACGGAAT	333	60
*RuBP*	FS083182	GCAGGTGTGGCCACCAATTAACAA	TGCACTCTCCGACCTCATTCAACA	269	60

**Table 8:** Primers used for real-time PCR expression analysis of six candidate reference genes

**Name**	**Forward primer 5′-3′**	**Reverse primer 5′-3′**	**Length (bp)**	**Tm (°C)**
*18S rRNA*	CGCGCGCTACACTGATGTATTCAA	TACAAAGGGCAGGGACGTAGTCAA	172	60
*APRT*	GAGATGCATGTAGGTGCTGTGCAA	GGCCCTTCAATTCTGGCAACTCAA	163	60
*GAPDH*	ATGGCCTTCAGAGTACCAACTGCT	GCTTGACCTGCTGTCACCAACAAA	189	60
*Cyclophilin*	GCGCCAAATTCAAGGACGAGAACT	ACAGCCTCGGCCTTCTTAATCACA	196	60
*Actin*	TGTGTTGGACTCTGGTGATGGTGT	TCACATCCCTGACGATTTCTCGCT	185	60
*RuBP*	TCGAGACTGAGCACGGATTTGTGT	TGCACTCTCCGACCTCATTCAACA	141	60

### DNA isolation

Young leaves of one month old plants were ground in liquid nitrogen and genomic DNA was extracted by using NucleoSpin plant II kit (Macherey-Nagel). The genomic DNA was quantified by using Nanodrop ND-1000 spectrophotometer (Thermo Scientific) and used for PCR.

### RNA isolation, quality controls and cDNA synthesis

Total RNA was extracted initially from the leaves for cloning the target genes and subsequently from various other plant tissues like young leaf, mature leaf, root, shoot, flower bud and open flower using Spectrum Plant Total RNA Kit (Sigma-Aldrich). The quality and concentration of each of the RNA samples was determined using Nanodrop ND-1000 spectrophotometer (Thermo Scientific), Bioanalyzer (Agilent 2100). Only those RNA samples having a ratio of 1.9 to 2.1 at 260/280; 2.0 to 2.5 at 260/230 and RIN (RNA integrity number) more than 7.0 were used for the analysis. The integrity of RNA was also checked by agarose gel electrophoresis. About 500 ng of each of the RNA was reverse transcribed using cDNA synthesis Kit (Superscript VILO, Invitrogen) according to the manufacturer’s instructions and used for further analysis.

### Cloning and sequencing of the housekeeping genes

*18s rRNA*, *APRT*, *GAPDH*, *Cyclophilin*, *Actin* and *RuBP* were PCR amplified from both the genomic DNA and cDNA of eggplant leaf; PCR amplification reactions were performed in 50 *μl* reaction volume containing 5 *μl* 10 x assay buffer, 200 *μM* each of dATP, dCTP, dGTP and dTTP (Fermentas), 0.5 *μM* each primer (Table 7), 0.5-2 units of Taq polymerase (Sigma-Aldrich) and 1 *μl* genomic DNA (50 ng)/cDNA. The PCR cycles consisted of initial denaturation at 94°C for 4 min, followed by 35 cycles of denaturation at 94°C for 60 s, annealing at 60°C for 30 s and extension at 72°C for 1 min with a final extension at 72°C for 10 min. The amplified products were later resolved on 1.2% agarose gel.

The amplified cDNA PCR products were cloned into pGEM-T Easy cloning vector (Promega) according to the manufacturer’s instructions. Freshly prepared competent cells of *Escherichia coli DH5α* were transformed with the recombinant plasmids. Positive clones were selected by blue white colony screening. Inserts in the clones were confirmed by restriction digestion with *EcoRI* and sequenced by ABI solid sequencing platform (Safelab).

### Real-time PCR

Quantitative Real-time PCR (qRT-PCR) was performed using SYBR Green I technology in Realplex^2^ thermal cycler (Eppendorf). A master mix for each sample was prepared with SYBR Green I, blue dye, ROX passive reference and stabilizers, PCR Core Reagents (Eurogentec). Reaction mix of 10 *μl* was prepared by adding 2.5 *ng* of cDNA and 750 *nM* each of the specific primers (Table 8). The amplification reactions were carried out at 95°C for 5 min, 40 cycles at 95°C for 15 seconds followed by 60°C for 1 min in qPCR high profile non skirted white 96-well plate (Eurogentec). Specificity of amplification was assessed by disassociation or melt curve analysis at 60-95°C after 40 cycles. Real time PCR analysis was carried out for three biological replicates for each sample and three technical replicates were analyzed for each biological replicate.

### Analysis of gene expression stability

Three statistical softwares; BestKeeper, NormFinder and geNorm were used for measurement of stability of expression of the six candidate genes.

### BestKeeper analysis

BestKeeper computed the gene expression variation for the six reference genes in all the samples based on crossing points (CP) [32]. Primary analysis of the qRT-PCR data based on the assessment of raw CP values calculated the standard deviation, SD (± CP) and coefficient of variance, CV (% CP) for the target genes in all the samples. This data was further used to determine the stability of gene expression. Based on the variability, control genes were ranked as the most stably expressed showing lowest variation to the least stable one with the highest variation. All the reference genes showing stable expression were combined into BestKeeper index for the individual sample using the geometric mean of the CP values for each of the candidate gene [15]. Samples with efficiency corrected intrinsic variation within three fold over or under expression were considered acceptable.

### NormFinder analysis

NormFinder utilizes a model based approach to establish expression stability of candidate reference genes. It uses raw data as an input in the form of expression values generated using the comparative Ct method. It estimates the overall expression variation of the candidate reference genes and the variation between sample subgroups [33].

### geNorm analysis

The geNorm was used to calculate candidate reference gene stability values (M) using raw expression data. Expression stability measure is calculated as the mean of pairwise variation of a gene compared to that of all other genes [34].

### Validation of expression of the identified reference genes

#### In other eggplant varieties

Seeds of four popular eggplant varieties, Pusa Shyamla, Pusa Ankur, Pusa Kranti and Pusa Uttam were procured from Division of Vegetable Science, Indian Agricultural Research Institute, New Delhi. Total RNA was extracted from 15 days old seedlings and used for cDNA synthesis, followed by expression analysis.

#### In transgenic eggplant

The selected genes were also validated in randomly selected lines of transgenic eggplant using the leaf tissue and compared with the wild type. Total RNA extracted from the leaf samples of transgenic plants was used for qRT-PCR.

#### Nematode infected eggplant

The shortlisted reference genes were also validated in nematode (*Meloidogyne incognita*) challenged eggplants. The roots of 15 days old plants were inoculated with approximately 300 freshly hatched infective second stage juveniles of *M. incognita*. The nematode inoculated plants were grown in a growth chamber at 27°C, 70% relative humidity and 16 hr light and 8 hr dark conditions for 30 days for the nematode to complete its lifecycle. Subsequently, the leaves were used for real time PCR to study the expression of the selected genes.

## Competing interest

The authors declare that they have no competing interest.

## Authors’ contributions

UR conceived and designed the experiments and contributed in the manuscript preparation. NPG performed the qRT-PCR, statistical data analysis and preparation of the manuscript. PP carried out the cloning of housekeeping genes and helped in script editing. PKT performed the statistical analysis and wrote the script. DK generated the transgenic and the nematode infected eggplant material. RS improved and carted the script. All authors read and approved the final manuscript.
